# High-Pitched Notes during Vocal Contests Signal Genetic Diversity in Ocellated Antbirds

**DOI:** 10.1371/journal.pone.0008137

**Published:** 2009-12-02

**Authors:** Yi-men Araya-Ajoy, Johel Chaves-Campos, Elisabeth K. V. Kalko, J. Andrew DeWoody

**Affiliations:** 1 Escuela de Biología, Universidad de Costa Rica, San José, Costa Rica; 2 Department of Biological Sciences, Purdue University, West Lafayette, Indiana, United States of America; 3 Institute of Experimental Ecology, University of Ulm, Ulm, Germany; 4 Smithsonian Tropical Research Institute, Balboa, Panama; 5 Department of Forestry and Natural Resources, Purdue University, West Lafayette, Indiana, United States of America; University of Sussex, United Kingdom

## Abstract

Animals use honest signals to assess the quality of competitors during aggressive interactions. Current theory predicts that honest signals should be costly to produce and thus reveal some aspects of the phenotypic or genetic quality of the sender. In songbirds, research indicates that biomechanical constraints make the production of some acoustic features costly. Furthermore, recent studies have found that vocal features are related to genetic diversity. We linked these two lines of research by evaluating if constrained acoustic features reveal male genetic diversity during aggressive interactions in ocellated antbirds (Phaenostictus mcleannani). We recorded the aggressive vocalizations of radiotagged males at La Selva Biological Station in Costa Rica, and found significant variation in the highest frequency produced among individuals. Moreover, we detected a negative relationship between the frequency of the highest pitched note and vocalization duration, suggesting that high pitched notes might constrain the duration of vocalizations through biomechanical and/or energetic limitations. When we experimentally exposed wild radiotagged males to simulated acoustic challenges, the birds increased the pitch of their vocalization. We also found that individuals with higher genetic diversity (as measured by zygosity across 9 microsatellite loci) produced notes of higher pitch during aggressive interactions. Overall, our results suggest that the ability to produce high pitched notes is an honest indicator of male genetic diversity in male-male aggressive interactions.

## Introduction

Variation in song production can affect individual fitness by means of mate choice and male-male competition [1]. Receivers assess the quality of conspecifics to maximize their own fitness, while senders produce songs in order to enhance their own reproductive success [2], [3]. This can result in conflicts of interest between senders and receivers as theory predicts that songs must be under constraints to be honest indicators of an individual's “quality” [4]–[6]. A signal may not necessarily be costly to produce yet it can be honest if it is physically constrained; such signals can therefore indicate phenotypical attributes of the signaler and provide direct information of individual's quality [7], [8]. Consequently, potential mates and rivals may use variation in the performance of constrained acoustic features to assess male quality [2].

The spectral and temporal features of vocalizations in birds are constrained by the limits of the vocal apparatus [9]. For instance, the production of one acoustic feature near its biomechanical limit can constrain the performance of other important vocal features [10]–[13]. In songbirds, such a tradeoff occurs, for instance, between element bandwidth and repetition rate due to biomechanical limitations [14]. The production of elements with larger bandwidth constrains repetition rate, whereas the production of elements with high repetition rate constrains element bandwidth. This tradeoff has important evolutionary implications, as it has been shown that females prefer those trilled songs that are closest to biomechanical limits [15], [16]. Furthermore, males sing songs closest to the biomechanical limits during aggressive interactions [17] as those repel male competitors more effectively [18].

The extent to which birds can push their biomechanical limits during song performance depends on different aspects of their phenotypic and/or genetic quality [6], [19]. Genetic diversity can directly affect song production through developmental instability of the song production system [20], and indirectly by affecting the individual's overall condition and therefore its ability to sing [21], [22]. If genetic diversity affects the development of the vocal apparatus and/or bird's body condition, song performance may be directly correlated with individual genetic diversity as a result. Previous studies have shown that genetic diversity (zygosity) of birds is associated with morphology [23] and song production [20], [22], [24]. In this context, biomechanically constrained acoustic features are increasingly seen as honest indicators of singer quality [11] where constrained acoustic features in songs are likely to honestly reveal genetic diversity, and potentially also the genetic quality of the singer. To our knowledge, this hypothesis has not yet been tested in the field.

Most studies on vocalization production in birds have been conducted in songbirds (i.e. suborder oscines within passerine birds), an avian clade in which vocalization production is believed to be mostly learned [25]. Although genetic diversity can potentially affect the development of the song production system or overall condition in birds, a link between vocalization performance and genetic diversity may be confounded by the learned component of song production in oscines. In contrast, vocalization production within the suborder suboscines has been poorly studied and is believed to be innate [25]–[27]. For this reason, the link between genetic diversity and acoustic features could be more direct in suboscine than in oscine birds.

**Figure 1:**
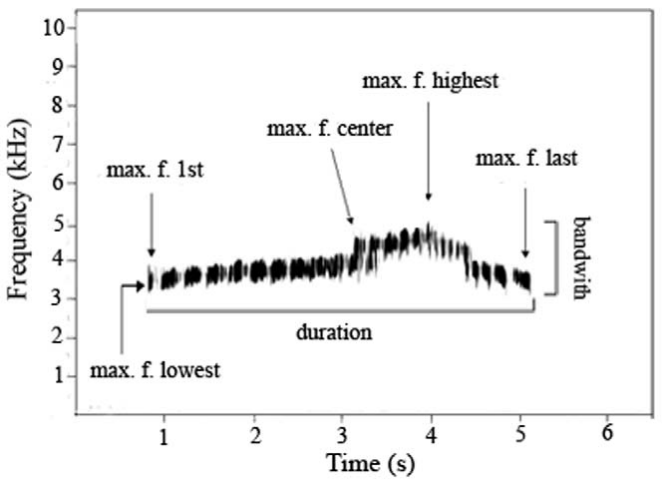
Annotated spectrogram of the “loud song” of a male ocellated antbird. Loud song structure was quantified using on-screen cursors to measure the following time and frequency traits: (1) maximum frequency of the first element (Max F. 1st) (2) maximum frequency of center elements (Max F. mid), (3) maximum frequency of the last element (F. last), (4) maximum frequency of the element with highest frequency (Max F. highest), (5) maximum frequency of the element with the lowest frequency (Max F. Lowest), (6) maximum frequency of the entire vocalization (Max F.)*, (7) total bandwidth of the vocalization (bandwidth), (8) vocalization duration (Duration), (9) time elapsed until the element with the highest frequency was produced (Time to Max F Highest )*, (10) time elapsed until the element with the maximum frequency of the entire vocalization was produced (Time to Max F )*, (11) number of elements produced after the element with the highest frequency*. Maximum frequency refers to the frequency with most energy in the element. * Measurements not shown in the annotated spectrogram.

We evaluated the link between vocalization performance and genetic diversity in a natural population of the ocellated antbird (*Phaenostictus mcleannani*: Thamnophilidae), a suboscine bird. Ocellated antbirds congregate at swarms of army ants to feed on arthropods that flee from the ants, and these birds produce relatively simple vocalizations [28]. We focused on a vocalization originally named “loud song”, which is produced year round by both sexes. It is used as a contact call between mates and in agonistic interactions between males [28]. The loud song is composed of stereotyped elements that rise in pitch, relative amplitude, and repetition rate at the beginning and center part of the song and decrease in pitch, amplitude and repetition rate at the end of the vocalization (Fig. 1).

We focused on vocalizations produced during male-male vocal contests and evaluated whether individual variation in the performance of constraint acoustic features in male ocellated antbirds reveal singers' genetic diversity, a proxy of genetic quality. If signals are used as a cue of sender quality, individuals must vary in their performance. Therefore, we first determined whether individual variation exists among males in different acoustic features of the loud song. We then tested for correlations between temporal and spectral features of the vocalization [13], [14], [29] to identify possible tradeoffs between acoustic features. After identifying acoustic features involved in tradeoffs, we used playback experiments to test whether males increase the production of these constrained features during aggressive interactions, as it has been shown in songbirds [17]. Finally, we tested the prediction that genetic diversity of individuals should be related to those acoustic features of the vocalization that appear biomechanically/energetically constrained and that are used during aggressive interactions.

## Methods

We studied a population of ocellated antbirds located in an area of 392 ha at La Selva Biological Station in Costa Rica (10°25′ N, 84°01′ W) [30]. Data were collected in three 14-week field seasons, from February 2005 to September 2006. Ocellated antbirds were captured with mist-nets set up at army ant colonies. A small blood sample was collected for molecular sexing and genetic analysis. All birds were uniquely banded. Some were fitted with a radio transmitter in each of the field seasons (for details on genetic analysis, tagging, and tracking see [30]). We obtained high-quality sound recordings (mean = 6 vocalizations per individual, range 4–10) from 13 individuals. The Organization for Tropical Studies (OTS) and the Costa Rican government granted permission to conduct this research (permits 013-2005-OFAU, R-CM-OET-05-2006-OT). We followed the ethical guidelines of the Purdue University Animal Care and Use Committee (permit number 04–024).

### Individual Variation in Vocal Features

We recorded vocalizations of known males during natural male-male aggressive interactions and during simulated aggressive dominance challenges (see below), using a ME 66 directional microphone (Sennheiser, Wedemark, Germany) with a Sony minidisk recorder (Sony Corporation, Tokyo, Japan). Songs were digitized uncompressed at a sampling rate of 44.1 kHz. Spectrograms were generated using Avisoft SASLabPro Version 4.0c (Avisoft Bioacoustics, R. Specht, Berlin, Germany). Spectrograms were produced using the broad-band (323 Hz) filter settings, and applying a flat top window, frame size 54%, overlap 87.5% and a Fast Fourier transformation of 1024. The spectrogram parameters resulted in a spectral resolution of 43 Hz and a temporal resolution of 3 ms. We measured several spectral and temporal variables from each spectrogram (Fig. 1) with a cursor on the screen.

We used analysis of variance (ANOVA) to evaluate variation among 13 males in each measured variable. We used “individual” as factor and “acoustic feature” as dependent variable. The assumptions of normality and homoscedasticity were met in all cases. All analyses were performed using R 2.7.1 [31] unless otherwise stated.

### Tradeoff between Vocal Features

**Figure 2:**
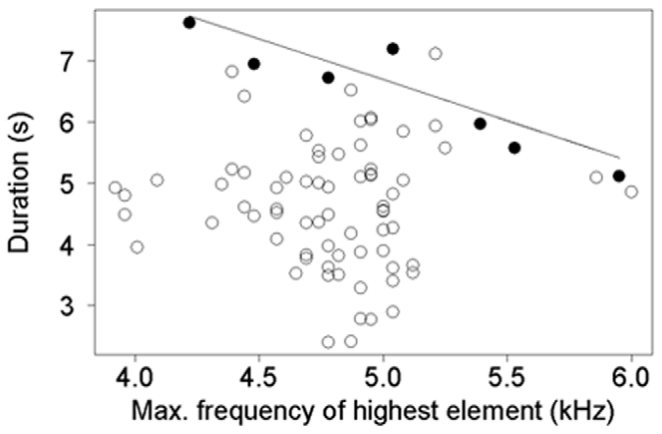
Correlation between the frequency of the element with highest pitch and vocalization duration in the “loud song” of ocellated antbirds. Filled circles show the values of maximum performance for seven 300-Hz bin according to an upper bound regression analysis (see text).

We tested for potential correlations between spectral and temporal vocal features of 13 males to identify possible biomechanical/energetic constraints. In one case, we used the “upper bound” regression method described in [14] to evaluate a potential tradeoff between vocal features in which the variance in one vocal feature depends on another vocal feature. In our particular case, individuals that sang at high frequencies did so for short periods only, whereas individuals singing at low frequencies did so for both short and long periods (Fig. 2). The upper bound regression method is increasingly used to analyze song performance (e.g. [13], [17], [29]). It consists of binning vocalizations into Hz increments of regular width, and selecting the vocalization with the maximum value of the feature of interest within each bin. Only the selected values are used in regression analysis, which allows analyzing only those vocalizations where song performance was maximized with regard to the features of interest. To evaluate the relationship between vocalization frequency and maximum vocalization duration, we binned songs into 300-Hz increments and selected the vocalization with the longest duration within each bin. The binning process resulted in seven bins, and therefore in seven vocalizations, which came from 6 individuals. We then used these seven vocalizations in a linear regression analysis that assessed a potential negative relationship between vocalization frequency and vocalization duration in vocalizations performed close to their maximum biological limit.

### Playback Experiments

We recorded loud songs from marked males that were singing spontaneously at army ant swarms, presumably representing contact calls between mates. These songs were compared with the vocalizations recorded after using playbacks to expose the same marked individuals on different days to simulated acoustic dominance challenges. Fifteen individuals were included in the analysis. Generally, ocellated antbirds show site-related dominance, whereby only the dominant bird in a given area responds aggressively to an acoustic challenge from either males or females [32]. In order to simulate aggressive challenges, playback experiments were conducted while radiotagged males were foraging at an ant swarm in their dominance areas. The stimulus was broadcast from a MA 840 speaker (frequency response from 70 Hz to 14 kHz; Polyplanar, Maryland, USA) located 10 to 20 meters from the focal individual and connected to a CD Player (SL-MP70, Panansonic, Osaka, Japan) with a mini-amplifier (Radioshack, Texas, USA). The aggressive stimulus consisted of loud songs from an individual different from the respective radiotagged males present at the ant swarm. Songs were digitized with no compression at a sampling rate of 44.1 kHz and standardized in the program Raven 1.3 to approximate the sound pressure level of field records (60 dB at 10 meters, measured with a Sper Scientific 840014 mini sound meter). Songs were broadcast in three one-minute periods, including five loud songs each. The one-minute vocalization periods were interspersed by one-minute intervals of silence. We alternated the vocalizations of five individuals among playback experiments to minimize pseudoreplication [33]. We used generalized linear mixed models in SAS 9.1 (SAS Institute Inc., North Carolina, USA) to test for differences in acoustic features between vocalizations produced in response to aggressive simulations and those recorded spontaneously. The behavioral context of the vocalization (interacting with mate, aggressive response) was considered as a fixed effect in the model, while the identity of the individual that produced the vocalization was included as a random effect to account for repeated measures of the same bird.

### Correlation with Genetic Diversity

We calculated individual values for each vocalization variable by taking the mean across replicates from the same individual. These individual mean values were used in a regression analysis with estimates of genetic diversity from DNA samples of 10 individuals. We calculated three widely used indices of individual genetic diversity (reviewed in [34]): internal relatedness (IR), homozygosity by loci (HL), and standardized heterozygosity (HS) using 9 polymorphic microsatellites developed for this population of ocellated antbirds, with a mean of 7.4 alleles per locus (additional details in [30]). By definition, HS should be negatively correlated with IR and HL.

Correlations between multilocus microsatellite zygosity and phenotypic traits can arise because one or more microsatellite loci are in gametic phase disequilibria with a functional gene that contributes to the phenotype in question (local effects), or because multilocus zygosity reflects genome-wide inbreeding (general effects) [35], [36]. We evaluated the possibility of local effects by considering differences in vocal features between heterozygotes and homozygotes in an iterative manner, one locus at a time, using binary logistic regression and student t-tests. The possibility of general effects was assessed by calculating the inbreeding coefficient (fixation index *F*_IS_) across loci in the population, thereby testing for a significant difference from zero (i.e. departure from a random union of gametes) by means of a randomization test in the program SPAGEDi 1.2e [37]. We also determined whether homozygosity was uniformly distributed across all markers as expected in case of genome-wide inbreeding [38]. We implemented the randomization test described in [38] using the program PopTools [39]. The test generated and compared two data sets derived from the entire data set of genotyped individuals. Each data set included either four or five randomly selected loci (without replacement) from the full set of 9 loci. We calculated IR (for simplicity) for each individual in each dataset and then correlated both datasets. This procedure was repeated 10,000 times to determine the mean value of the correlations which should be positive in case of genome-wide inbreeding [38].

## Results

### Individual Variation in Vocal Features

We recorded loud songs from 13 adult males during aggressive interactions in the field to evaluate individual variation in selected acoustic features (Fig. 1). As predicted, we found statistically significant variation among individual males (Table 1). Most individuals differed in acoustic features between each other during aggressive song contests.

**Table 1:** ANOVA results for individual variation in the aggressive vocal features of ocellated antbirds.

Acoustic feature	F_12,79_
Max F. 1st (kHz)	4.52
Max F. last (kHz)	3.97
Max F. center (kHz)	7.90
Max F. highest (kHz)	10.88
Max F. lowest (kHz)	3.72
Max. F (kHz)	11.04
Bandwidth (kHz)	4.84
Duration (kHz)	5.25
Time to Max F. Highest (s)	6.13
Time to Max F (s)	4.18
Number of elements after Max F highest	4.57

See Fig. 1 for definition of acoustic features. All tests were significant (P<0.001 in all cases). Most individuals differed from each other for each acoustic feature according to posthoc Tukey tests.

### Tradeoffs between Vocal Features

Individuals that sang at high frequencies did so for short periods only, whereas individuals singing at low frequencies did so for both short and long periods. We used an upper bound regression technique to analyze this particular case and discovered a strong negative relationship between vocalization duration and frequency (regression equation, variable1 =  slope x variable2+ intercept; duration  = −1.39 x frequency +13.49, R^2^ = 0.84, F_1,5_ = 31.97, p = 0.002; Fig. 2). Longer songs were associated with lower frequencies and shorter songs with high-pitched notes. This is consistent with the idea that high frequencies cannot be sustained over longer periods, resulting in a tradeoff between temporal and spectral aspects of the vocalization.

### Playback Experiments

We compared vocalizations recorded from males in mate-mate interactions with responses to simulated challenges of dominance using playbacks. In comparison with loud songs directed toward mates, loud songs recorded during playbacks were higher in pitch except for the frequency of the lowest element and the frequency of the final element. Males also produced longer vocalizations and reached the maximum frequency faster when responding to playbacks (Table 2). We interpret the responses of the male ocellated antbirds as aggressive because all individuals flew towards the speaker and performed aggressive displays as described in [28].

**Table 2:** Variation in acoustic features of ocellated antbirds between loud songs directed towards mates and loud songs elicited by an aggressive acoustic stimulus.

	Mate		Aggressive		Tests	
Acoustic feature	Mean	SD	Mean	SD	F	P
Max F. 1st (kHz)	2.95	0.07	3.09	0.07	5.87	0.01
Max F. last (kHz)	3.16	0.07	3.07	0.06	1.28	0.26
Max F. center (kHz)	3.72	0.10	4.03	0.10	21.21	<0.01
Max F. highest (kHz)	4.55	0.10	4.8	0.90	19.88	<0.01
Max F. lowest (kHz)	2.68	0.07	2.66	0.06	0.18	0.67
Max F. (kHz)	3.69	0.10	3.9	0.09	10.27	<0.01
Bandwidth (kHz)	1.86	0.08	2.16	0.07	20.3	<0.01
Duration (s)	3.81	0.25	4.74	0.22	19.18	<0.01
Time to max F. highest (s)	2.87	0.14	3.13	0.13	5.34	0.02
Time to max F. (s)	2.37	0.19	1.82	0.16	10.67	<0.01
Number of elements after max F. highest	1.24	0.36	3.04	0.3	30.13	<0.01

F =  ANOVA F statistic; degrees of freedom for each test = 1,105. See Fig. 1 for definition of acoustic features.

### Correlation with Genetic Diversity

**Figure 3:**
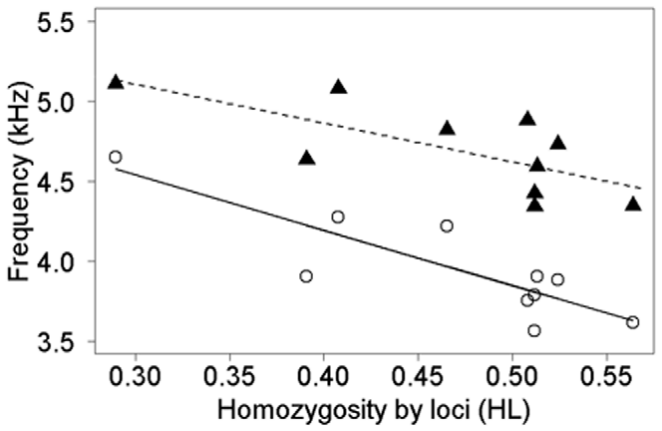
Correlation between song features with genetic diversity (measured as homozygosity by loci) in ocellated antbirds. Song features: fundamental frequency of the center element (open circles, solid line) and fundamental frequency of the highest element (filled triangles, dashed line).

We found significant relationships between individual genetic diversity and two spectral variables recorded during aggressive vocalizations. First, there was a strong relationship between genetic diversity and the maximum frequency in the central element of the vocalization; frequency increased with an increase in genetic diversity. Note that increases in diversity correspond to increases in HS (frequency  = 1.39 x HS + 2.77, R^2^ = 0.49, F_1,8_ = 9.53, p = 0.01) and decreases in IR and HL (IR: frequency  = −2.90 x IR + 5.15, R^2^ = 0.50, F_1,8_ = 9.87, p = 0.01; HL: frequency  = −3.45 x HL + 5.57, R^2^ = 0.69, F_1,8_ = 21.43, p = 0.002). We only plotted frequency vs. HL for simplicity (Fig. 3). Second, we found similar relationships between genetic diversity and the maximum frequency of the highest element in the vocalization, but only the relationship with HL was statistically significant (HS: frequency  = 0.91 x HS + 3.92, R^2^ = 0.25, F_1,8_ = 3.96, p = 0.08; IR: frequency  = −1.91 x IR + 5.48, R^2^ = 0.26, F_1,8_ = 4.17, p = 0.08; HL: frequency  = −2.42 x HL +5.83, R^2^ = 0.45, F_1,8_ = 8.38, p = 0.02). Together, these patterns suggest that increased genetic diversity is associated with the production of higher frequencies.

Because multilocus patterns can be driven by a strong effect at one or a few loci (e.g., those tightly linked to relevant genes), we tested the idea that zygosity at individual loci is sufficient to influence song structure, using either logistic regression or t-tests. We did not find significant differences in song structure between homozygotes or heterozygotes at any single locus (results not shown), which suggest that our results are more likely to be the product of a general rather than a local effect.

We did not detect, however, a genome-wide effect of inbreeding using the test suggested by [38]. The two randomly chosen subsets of loci were in fact negatively correlated (mean correlation ± standard deviation: −0.63±0.15). This is consistent with our results from the fixation index (*F*_IS_), which indicates that mating is random with respect to relatedness (*F*_IS_ = 0.01; p = 0.73; permutation test; n = 57 individuals).

## Discussion

Our research led us to four major insights regarding acoustic communication in male ocellated antbirds. First, the highest frequency produced in aggressive vocalizations varies between individuals. Second, high-pitched vocalizations are difficult to sustain over time, presumably because of high energetic costs and/or biomechanical constraints. Third, the pitch and duration of vocalization increases during aggressive interactions. Finally, individuals with greater genetic diversity produce notes of higher pitch. Overall, these results show that the ability to “hit the high notes” during loud songs in ocellated antbirds correlates with male genetic quality, and that these high pitched notes are used during ritualized aggressive interactions mediated by vocalizations.

### Individual Vocalization Performance and Tradeoff between Variables

As suboscine birds such as ocellated antbirds lack a learned component in their vocalizations [25], [26], variation detected in the loud songs among individuals must be caused by genetic and/or epigenetic individual differences linked to specific features of the nervous system, vocal anatomy, metabolic, and/or physical condition [6]. Furthermore, a negative correlation between duration and highest frequency of the vocalization was revealed by the upper bound regression (Fig. 2). We interpret this result as a tradeoff between frequency and duration caused by constraints in song production, similar to the tradeoff between bandwidth and repetition rate that has been found in songbirds [11], [14]. These results are consistent with the possibility that individual variation is partially driven by differences in the ability to push biomechanical and/or energetic limits of the vocal apparatus.

The parts of the song with high-pitched notes also have the highest repetition rate (Fig. 1), and although the mechanism of sound production in suboscine birds is poorly understood, it is possible that the tradeoff between vocalization frequency and duration could be explained by individual differences in respiration performance. In oscines, the tradeoff between element bandwidth and repetition rate could be explained by individual differences in respiration performance; specifically, the production of trills leads to a net loss of respiratory volume [40]. The anatomy of song production has not been studied in the ocellated antbird but it has been studied in other suboscines. In the Great Kiskadee (*Pitangus sulphuratus*; Tyrannidae), the pressure in the air sac needed during exhalation increases with vocalization frequency [41]. In addition, it is known that the basic temporal pattern of a song in birds is determined by their respiratory rhythm [42]. Birds exhale during song production and inhale during brief periods between syllables to prevent the depletion of their air supply; however, a net loss of respiratory volume occurs during the production of fast notes [40]. Ocellated antbirds produce high pitched notes at a very fast rate (Fig. 1), which suggests that they exhale more air than they can recover during the very brief periods of inhaling between notes. We therefore propose that ocellated antbirds experience a loss of respiratory volume during the production of high pitched notes that ultimately results in a constraint between the total duration and the frequency of the vocalization. Given these respiratory demands in the production of high pitched notes, we hypothesize that only high quality males can produce vocalizations of high pitch and long duration. These hypotheses warrant experimental evaluation under laboratory conditions with suboscine birds.

### Variation in Vocal Features during Playback Experiments

Our experimental data provides evidence of the importance of high pitched notes and vocalization duration during male-male vocal contests. Male ocellated antbirds produced vocalizations that were of higher pitch and longer duration during responses to aggressive challenges compared to situations where they sang spontaneously to contact their mates (Table 2). Given the trade-off outlined above, it is possible that male ocellated antbirds use specific acoustic traits, particularly high pitched notes combined with vocalization duration to assess the quality of contenders [2]. We hypothesize that males signal their quality by performing vocalizations as close to their biomechanical limits as possible, i.e. by increasing the pitch and the duration of the vocalization as much as they can given the tradeoff between these variables.

### Correlation of Vocal Characteristics with Genetic Diversity

In our study, we found a strong correlation between vocal characteristics and genetic diversity. Generally, genetic diversity was higher in birds that produced higher-pitched notes. Potentially, song characteristics of male ocellated songbirds can be taken as indicators for individual fitness as reduction or lack of genetic diversity may indicate reduced individual fitness due to increased homozygosity [43]. This can happen either by expression of deleterious recessive alleles or by reduction of heterozygote advantage [44]. In theory, either possibility could explain correlations between song traits and genetic diversity [20], [22], [24]. If genetically diverse individuals experience less physiological stress or developmental instability in their vocal apparatus, a correlation between genetic diversity and the ability to produce high-pitched notes could arise in ocellated antbirds. Alternatively, given that heterozygosity can affect overall body condition [45]–[47], birds in better body condition should produce songs of higher pitch. As these two mechanisms are not mutually exclusive, the proposed association of genetic quality of males with song characteristics might be due to multiple proximate causes.

There are many empirical examples where heterozygosity has been implicated to influence performance, development, or some other aspect of fitness (reviewed in [35], [43]). These heterozygosity-fitness correlations, or HFCs, are often caused by direct effects whereby the marker locus, typically a gene or enzyme, directly influences fitness through its functional properties or due to local effects whereby the marker is tightly linked to a functional locus. Our locus-by-locus analyses provided no evidence for local effects of heterozygosity on vocal characteristics in ocellated antbirds.

Alternatively, HFCs may arise because of general, genome-wide effects not caused by inbreeding (e.g., heterosis). For example, surveys of over 1000 marker loci have revealed strong relationships between zygosity and disease traits in humans [48]. Although general HFCs are more difficult to detect with a small number of markers [36], [43], effects like those observed in our current study have already been documented in empirical studies [35], [49], including associations between genetic diversity and song variation in birds [20], [22], [24]. Moreover, as the avian genome size is generally smaller than other vertebrates [50] this may provide somewhat more power to detect general HFCs in birds. Furthermore, recent evidence suggests that general HFCs are highly susceptible to environmental conditions [51]. Overall, this may explain why HFCs are observed in some studies but not in others.

### Evolutionary Implications

Our data suggest that the ability of male ocellated antbirds to produce high-pitched vocalizations is constrained and is linked to genetic diversity. In songbirds, females prefer songs that are closest to biomechanical limits [15], [16]. If female ocellated antbirds also prefer males that sing at highest pitches, this could have evolutionary implications for the maintenance of genetic variance associated with sexually selected traits. Directional female mate choice is theoretically expected to deplete additive genetic variation (i.e. variance attributed to the average effect of substituting one allele for another) in male traits, and multiple hypotheses have been proposed to explain why this is not actually observed in nature [52]. If female ocellated antbirds prefer to mate with males that sing higher pitches, they would be also mating with heterozygous males rather than males that carry specific alleles [53]. This can prevent the depletion of additive genetic variance in males [54], which would maintain the variance of a trait even under the influence of directional selection, thus ultimately allowing individual assessment of male quality.

Finally, our results from ocellated antbirds may apply to other vertebrates. There is evidence from roosters that high fundamental frequencies are related to dominance status and are used during ritualized aggressive interactions among roosters [55]. Moreover, warblers that sing with high and consistent pitches are preferred as extra-pair mates [56], which suggest that high frequencies can also reveal the singer's quality in other species. It also has been suggested, however, that low frequencies reveal a singer's quality in birds and frogs, presumably because the production of low frequencies over high frequencies is mostly constrained by body size, weight or condition [8], [57]–[62]. Based on our study, we hypothesize that, at least in some species, the high frequencies in a song provide information about the singer's genetic quality that is not necessarily constrained by its body size or weight. Overall, given that vertebrates share some basic neuromuscular mechanisms of sound production, the use of high pitched notes to assess the quality of possible mates or rivals could be a widespread phenomenon.
